# Scale Norming Undermines the Use of Life Satisfaction Scale Data for Welfare Analysis

**DOI:** 10.1007/s10902-021-00460-8

**Published:** 2021-10-12

**Authors:** Mark Fabian

**Affiliations:** Department of Politics and International Studies, The Bennett Institute for Public Policy, University of Cambridge, Alison Richard Building, 7 West Road, Cambridge, CB3 9DT UK

**Keywords:** Subjective well-being, Welfare analysis, Life satisfaction, Adaptation, Scale norming, Response shift, I3, I31

## Abstract

Scale norming is where respondents use qualitatively different scales to answer the same question across survey waves. It makes responses challenging to compare intertemporally or interpersonally. This paper develops a formal model of the cognitive process that could give rise to scale norming in year on year responses to life satisfaction scale questions. It then uses this model to conceptually differentiate scale norming from adaptation and changes in reference points. Scale norming could make life satisfaction responses misleading with regards to the changing welfare of individuals. In particular, individuals who would say that their life is "improving" or "going well" might nonetheless give the same scale response year after year. This has negative implications for the use of scales in cost–benefit analysis and other welfarist applications. While there is already substantial empirical evidence for the existence of scale norming, its implications for welfare analysis are sometimes understated on the grounds that this evidence might simply be the product of errors of memory. The paper presents new empirical evidence for scale norming from two surveys (N1 = 278; N2 = 1050) designed such that errors of memory are an unconvincing explanation for the results.

## Introduction

Scale norming is where the qualitative meaning of the points on a respondent’s scale changes between survey waves (Frederick & Loewenstein, 1999). Stillman et al.’s (2015) study of Tongan migrants to New Zealand provides an illustrative example of scale norming. New Zealand uses a lottery system to allocate visas to would-be migrants from Tonga. Around 10% of applicants are successful and migrate to New Zealand, where they typically experience meaningful increases in their real incomes. Stillman et al. survey both successful and unsuccessful applicants 2–5 years after the lottery. One survey question is the ‘welfare ladder’, as follows:

Please imagine a 10-step ladder where on the bottom, the first step, stand the poorest, and on the highest step, the tenth, stand the rich. On which step are you today?

This is a scale question where the meanings of ‘poor’ and ‘rich’ and the intervening points on the ladder are subjective and need to be defined through a cognitive exercise when answering the question. In addition to this ‘welfare ladder’ question, the successful applicants (migrants) were asked which step of the ladder they were on when they were last living in Tonga. Despite their objectively higher on average real incomes, the migrants report being no higher on the welfare ladder than the unsuccessful applicants. However, they report being 0.75 rungs higher on average than they were when last living in Tonga. In other words, they seem to think that they are better off now than before, yet they give the same scale response.

At first, this may seem like a straightforward case of changing reference points. While the migrants are wealthier in New Zealand, they are also surrounded by wealthier people. They consequently adjust their evaluation of their old life downward. But imagine if the question were phrased differently: “are you on a higher step now than you were previously?”. The answer is surely yes. This improvement is obfuscated by the fact that the reporting scale used shifts between waves of the survey. The scale response doesn’t change, but the scale does, masking a real improvement in subjectively-assessed welfare. This is scale norming. Reference point changes (Easterlin & Plagnol, 2008), adaptation (Luhmann et al., 2012), and scale norming are hard to tease apart, as discussed in Sect. 2, below.

Consider how the migrants’ reporting on the welfare ladder would play out if it were a life satisfaction ladder instead. Assume that rather than increases in real incomes, the migrants had experienced increases in *latent* life satisfaction. However, they had also discovered new life satisfaction possibilities in New Zealand, which causes them to reconceptualise their scale. The outcome is then equivalent: *latent* life satisfaction increases by 0.75 steps, but the scale response doesn’t change, the scale itself changes instead. If you asked the migrant “are you more satisfied with life now than you were in Tonga?” the answer would again be yes.

This is important for welfare economics. Some scholars advocate for life satisfaction scale data to be our principle way of measuring social progress (Clark et al., 2018; Diener & Seligman, 2004). This seems fraught if a substantial portion of improvements in latent life satisfaction do not show up in the data because of scale norming. The phenomenon is even more pernicious to cost–benefit analysis using life satisfaction scale data, which some scholars have advocated for (Frijters et al., 2019; Fujiwara & Dolan, 2016). Cost–benefit analysis relies on precise, cardinal, interpersonal welfare comparisons (Adler, 2013). If the welfare effects of some changes are more or less susceptible to scale norming, or if certain groups are more or less prone to scale norm, then this will inject bias into measures and distort the conclusions of cost–benefit analyses.

There is substantial empirical evidence for the existence of scale norming (Schwarz & Sprangers, 2000), but it does not seem to be widely appreciated as a problem among subjective well-being scholars. This is perhaps because of a tendency to see patterns in longitudinal life satisfaction data that could represent scale norming as evidence of various cognitive biases instead. For example, the first four waves of the German Socio-Economic Panel (GSOEP) included a scale question for present satisfaction and a second question for satisfaction in the previous year. The correlation between the retrospective question and the actual response in the previous year is only 0.5. Similarly, waves 16 and 17 of the British Household Panel Survey (BHPS) include both a life satisfaction scale question and the following: “would you say you are more satisfied with life, less satisfied with life, or feel about the same as you did a year ago?”. This is a straightforward question about whether life is better now than before. The correlation between this question and life satisfaction scale responses is only 0.22. These data points may indicate scale norming, but subjective well-being scholars tend to interpret them instead as evidence for recall bias or errors of memory (Prati & Senik, 2020), effort justification (Howard & Dailey, 1979), and implicit theories of change (Norman, 2003).

This behavioural economic lens needs to be tempered with a more cognitive psychological perspective. Subjective well-being scholars perhaps assume that life satisfaction questions are cognitively simple to answer because respondents do so quickly (Diener et al., 2013; Stone & Mackie, 2013). Yet speed of cognition tells us little about complexity. Assessing one’s latent life satisfaction and then reporting it on a scale in a way that is consistent over time is potentially very difficult. This cognitive process is largely untheorized and has received limited empirical investigation (McClimans et al., 2012). Analysing the cognitive complexity of answering life satisfaction scale questions, as this paper does, reveals that scale norming is just as valid an interpretation of many patterns in life satisfactions data as cognitive biases in responding. Indeed, many of the claimed biases in responding may instead be a function of the measurement instrument rather than human cognition. Notably, if latent life satisfaction is not on a scale from 1 to 10 then asking people to report on such a scale requires them to contort their answers in some way, especially over multiple surveys. Such bias-by-instrument rather than cognition is what is implied, though not conclusively demonstrated, by the Stillman et al. (2015) study of migrant assessments of wealth, which can be measured objectively, unlike life satisfaction. It is quite possible that respondents are communicating with researchers as effectively as they can given the constraints of life satisfaction scale metrics, but a “bias bias” (Gigerenzer, 2018) on the part of researchers prevents respondents from being heard effectively.

This paper advances the scholarship of scale norming in two separate ways. The first half of the paper provides *conceptual clarification*. It further develops Fleurbaey and Blanchet’s (2013) model of what Oswald (2008) calls the ‘reporting function’. This is the cognitive process that maps latent life satisfaction into a numerical response on a life satisfaction scale. This model gives us a sense of what goes on, cognitively and linguistically, when respondents answer life satisfaction scale questions and illuminates how these processes could give rise to scale norming. The paper uses the model to conceptually distinguish scale norming from adaptation and reference point effects. The paper then reviews empirical evidence for the existence of scale norming, principally from the vignettes and response shift literature. The possibility of scale norming is often difficult to disentangle from other cognitive phenomena associated with subjective reports, notably recall bias, implicit theories of change, and effort justification. The paper explains why the latter two of these phenomena are fundamental to the reporting function and thus not good reasons to dismiss concerns about scale norming. This leaves recall bias as the main alternative explanation for trends in life satisfaction data that might indicate scale norming.

The second half of the paper provides *new empirical evidence* for the existence of scale norming from an experiment that is substantially robust to errors of memory critiques. Results from two samples (N1 = 277; N2 = 1050), one purposive and the other representative, both indicate widespread scale norming. The results suggest that individuals can say that their life is getting better even when their year-on-year life satisfaction responses are identical. This affirms the concern outlined above that scale norming can obfuscate subjectively-assessed improvements in life satisfaction over time, with pernicious implications for welfare analysis. This experiment is not a test of the conceptual framework presented in the first half of the paper. However, it repudiates the claim that scale norming is not worth investigating further as it can easily be explained as an error of memory.

## The Reporting Function

The ‘reporting function’ is a cognitive process that translates latent life satisfaction into a response on a scale question (Oswald, 2008). To get a handle on it, imagine two high school teachers, one considered a ‘generous’ grader and the other more ‘stringent’. It’s possible that both teachers assess the quality of a student’s work and rank it relative to classmates identically, but nonetheless assign different *grades* to that work. Similarly, people could have generous or stringent reporting functions and consequently communicate their identical assessments of life satisfaction differently on a scale instrument.

Pavot and Diener (1993) gave one of the first informal descriptions of the reporting function. They describe the process of making a life satisfaction evaluation as involving the individual constructing a “standard” that they perceive as appropriate for themselves, and then comparing the circumstances of their life to that standard. Fleurbaey and Blanchet (2013) developed a more formal and sophisticated model of “subjective well-being as it can be retrieved with typical questionnaires” (pg. 175).

Fleurbaey and Blanchet’s model takes the following form. They begin with a vector, ${{\varvec{l}}}_{{\varvec{i}}}$, that covers “the diversity of states, activities and possibilities enjoyed or endured by an individual [i] over the course of their life” (p. 175). When someone answers a life satisfaction questionnaire, they consider ${{\varvec{l}}}_{{\varvec{i}}}$ and there is some function, ${\xi }_{i}$, that maps this vector, including the individual’s actual life, ${l}_{i}^{*}$, into possible responses to the scale question, ${r}_{i}$. As Fleurbaey and Blanchet explain:

ξi(*l*_*i*_) must lie in a given scale, which can be a verbal scale (e.g. very satisfied/fairly satisfied/not very satisfied/not at all satisfied), or a numerical scale (e.g. from 0 to 10). The cognitive problem for the individual is to put the many dimensions of *l*_*i*_into one of a few ordered categories.

The reporting function maps the entire vector ${{\varvec{l}}}_{{\varvec{i}}}$ into the entire scale,${r}_{i}$. So we can let ${r}_{it}\in \{1, 2, ... , k, ... , K\}$ denote the choice of response category to a single life satisfaction scale survey item with *K* response options. However, the individual’s own life,${l}_{i}^{*}$, maps into a single response category,${r}_{i}^{*}$. The individual’s response to a life satisfaction question is thus given by${r}_{i}^{*}={\xi }_{i}({l}_{i})$. The challenge for the respondent is to communicate the complexity of their life satisfaction assessments,${{\varvec{l}}}_{{\varvec{i}}}$, within the constraints of the scale instrument. The challenge for researchers is to infer ${l}_{i}^{*}$ relative to ${{\varvec{l}}}_{{\varvec{i}}}$ from ${r}_{i}^{*}$ despite the messiness introduced by the reporting function. Fleurbaey and Blanchet identify three separate but related problems that are likely to plague such exercises: the scope, ranking, and calibration problems.

The scope problem concerns what aspects of *l*_*i*_ are relevant for the individual to consider. For example, what time frame is appropriate—today, this week, the time since the last survey? Should the state of the household be considered or just the individual? What about the state of the world in general?

The scope problem would give rise to scale-norming if respondents used inconsistent scopes across survey waves. When surveyed at the end of the financial year in wave 1 they may focus on their business, for example, whereas when surveyed around Christmas in wave 2 they might focus on their family. The points on the scales used in these two waves will have widely divergent meanings, which makes comparing responses on them questionable.

**Fig. 1:**
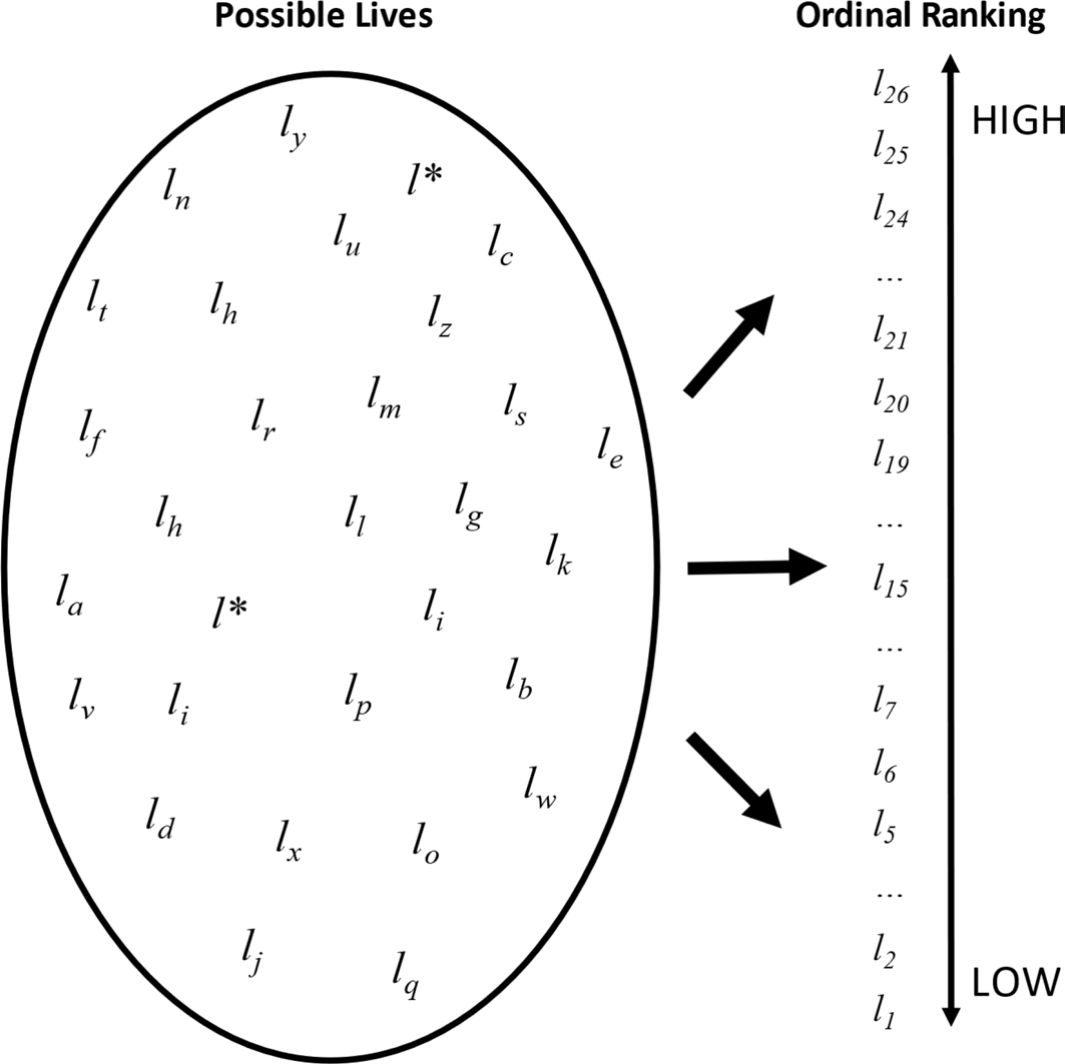
The ranking problem. *Notes*: Respondents must arrange possible lives they could live, of which there are theoretically an infinite number, into an ordered ranking from least to most satisfying

Where the scope problem is about what to consider when assessing one’s life satisfaction, the ranking problem is about how to weight those various considerations against each other. It is cognitively difficult to arrange relevant possible lives (e.g. 1_a_, 1_b_, etc.), including the individual’s actual life, *l*_i_*, into an ordinal pattern (e.g. 1_1_–1_9_), especially as there are many possible lives to consider. This is represented graphically in Fig. 1.

The complexity of the ranking exercise may induce respondents to focus on salient aspects of their immediate situation and forget other relevant dimensions of their life. The ‘focusing illusion’ is a famous example of this (Schkade & Kahneman, 1998). Respondents may focus on, say, the poor weather in their area and fail to take into consideration relatively low local living costs. SW-B scholars hope that avoiding explicit primes through smart survey design will mitigate this issue (Stone & Mackie, 2013). An example of an explicit prime is given by Deaton and Stone (2016). They found that asking about politics prior to life satisfaction reduces life satisfaction responses. However, it’s unclear that removing explicit primes negates the ranking problem. Even without external primes, respondents will frame their life in terms of whatever is bothering them at the time of the survey. These preoccupations may differ between surveys, giving rise to scale norming. For example, if the survey is taken during a difficult month at work, those difficult circumstances could crowd out the good salary, colleagues, and commute that dominate work assessments on the other 11 months of the year. During the good work months, the qualitative meaning of the points on the respondent’s scale might be determined by household issues instead.

**Fig. 2:**
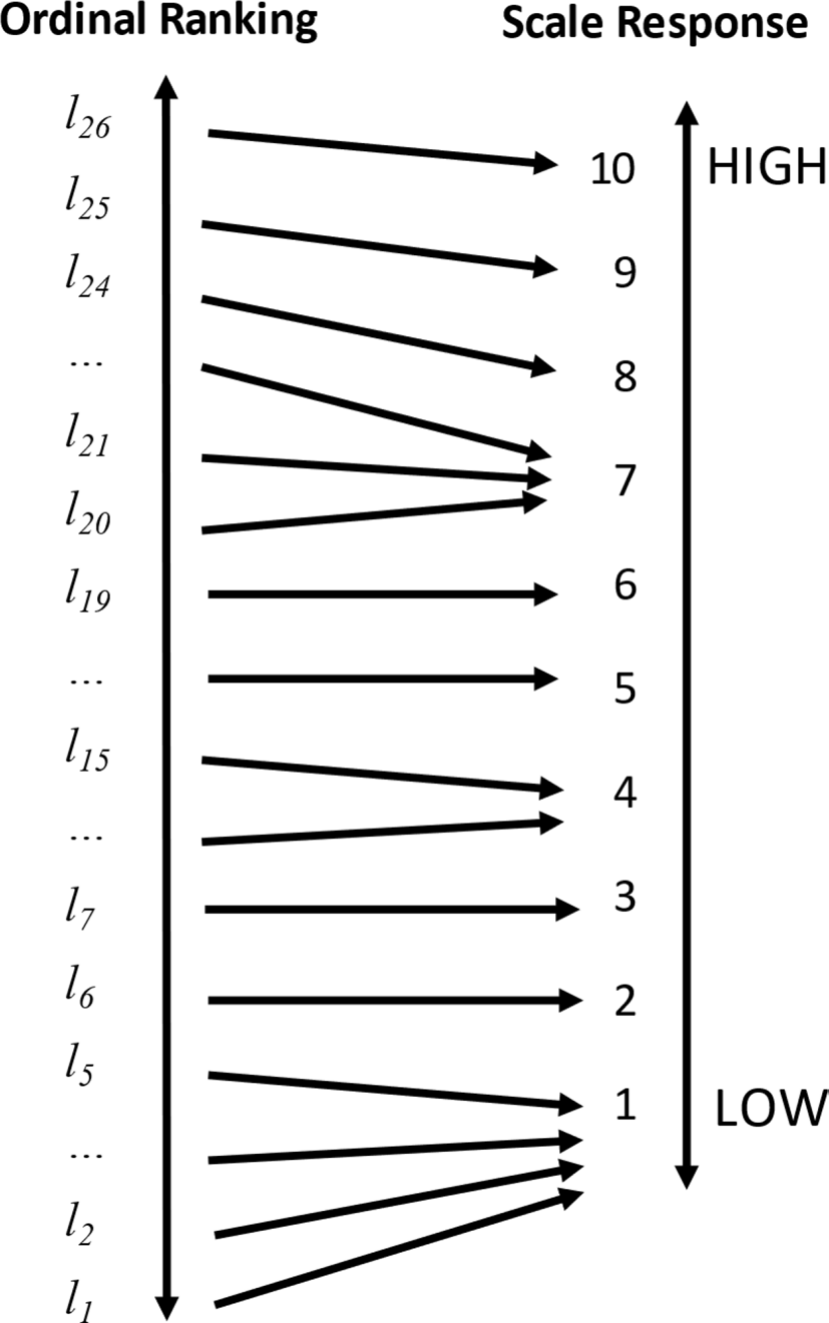
The calibration problem. *Notes*: The calibration problem involves mapping the many possible lives individuals might consider when evaluating their own life into the limited number of response categories on the scale instrument

The calibration problem is about how the individual maps their ranking of possible lives, including their own life, ${l}_{i}^{*}$, into the limited number of response categories available on the survey question. It is depicted graphically in Fig. 2. There is a strong framing effect present that arises out of the fact that the scale offered is closed. This contrasts with real life, where many considerations relevant to life satisfaction are open, like income, or have fuzzy limits, like career progression. The closed scale forces respondents “to move from reasoning in terms of life content to a reasoning in terms of a statistical distribution” (Fleurbaey & Blanchet, 2013, p. 181). The calibration problem is which distribution to choose. For example, should the respondent choose from among the lives available to all humans, including Lebron James, or rather from among those that seem realistically possible for themselves? Different calibrations across respondents and over time could create arguably incomparable responses.

An empirical example of the calibration problem comes from Ubel et al. (2005). They tested how respondents assess their health relative to “perfect health” when explicitly asked about perfect health for “a 20-year-old” or “someone your own age”. They found that unprimed respondents interpret perfect health somewhere between perfect health for a 20-year-old and someone their own age. As people age, this scale meaningfully changes such that an 80-year old has a different scale in mind than a 40-year old. A corollary point is that an 80-year old’s response scale would differ from the scale they used when they were 60.

**Fig. 3:**
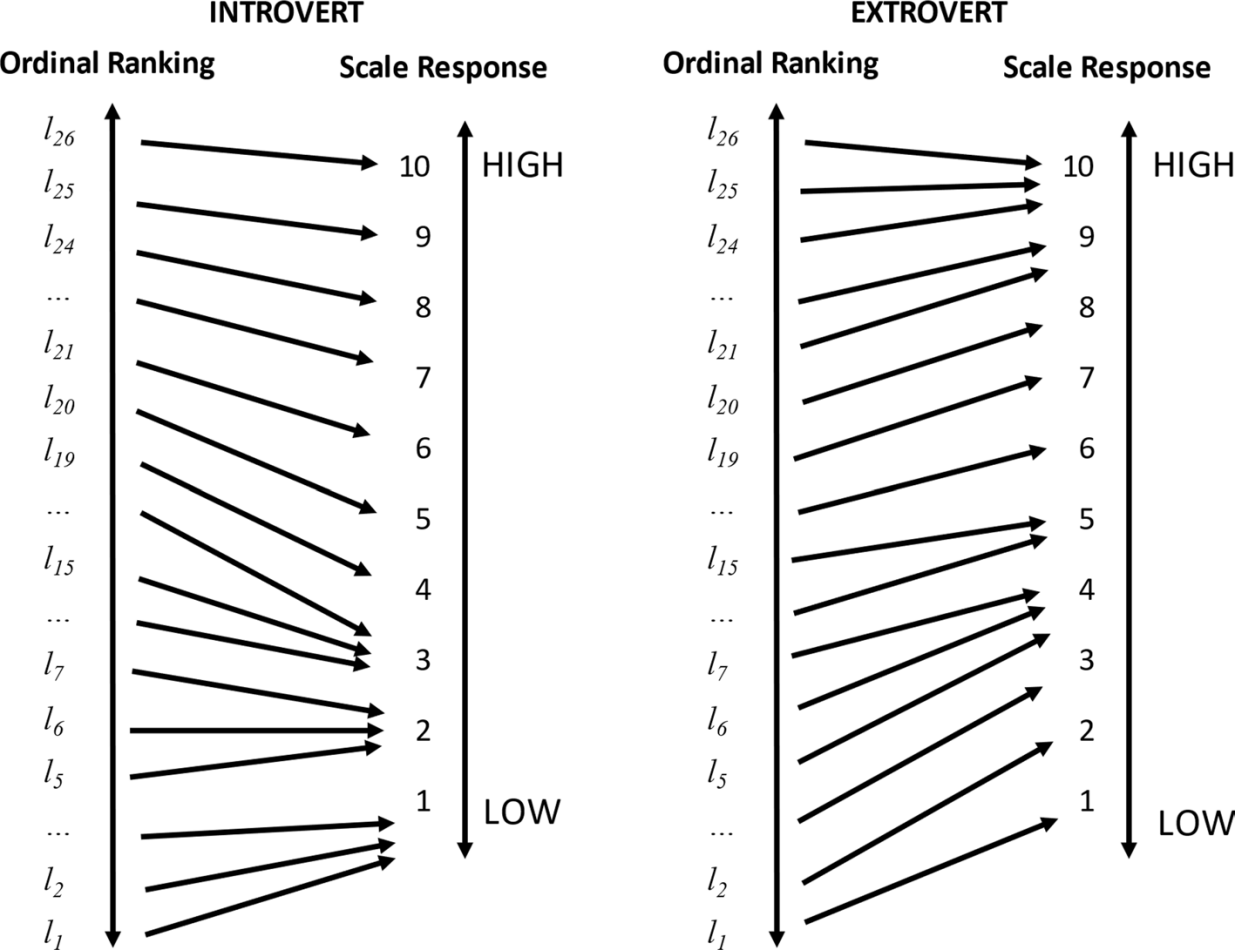
Differences in calibration styles, e.g. between introverts and extroverts. *Notes*: individuals who differ in their calibration style (for example, stringent or generous) may map identical subjective life satisfaction assessments into different response categories

The calibration problem is one explanation for why introverts and extroverts differ in their life satisfaction, on average (Diener & Lucas, 1999). Consider two individuals, A and B. Assume that they have identical *latent* life satisfaction, but A is introverted while B is extroverted. If this personality difference only affects reporting (by a positivity bias among extroverts, for example) and not latent life satisfaction (by extroverts having more friends, for example), then A would map their latent life satisfaction more stringently into lower responses to a scale question than B, who maps relatively generously. This is depicted graphically in Fig. 3.

Both A and B make the same assessment of their wellbeing; they just *report* it differently. Our measurement of their SWB is biased by our inattention to the reporting function. To understand why this is pernicious to interpersonal welfare comparisons, consider the following: assuming the difference between introverts and extroverts is purely a matter of reporting style, would the world have more wellbeing if all introverts were somehow converted to extroverts? More pointedly, should more policy effort be directed at introverts given their lower scale reports? The answer to both questions seems intuitively to be no.

To summarise, the problem with life satisfaction scales is that we want to know about ${l}_{i}^{*}$ relative to ${{\varvec{l}}}_{{\varvec{i}}}$**,** but we can only ever observe ${r}_{i}^{*}=\xi ({l}_{i})$. Furthermore, both *l*_i_* and ξi(*l*_*i*_) could change over time such that responses to life satisfaction scale questions become neither inter-temporally nor inter-personally comparable, even ordinally. This may go some way to explaining why scale responses have low test–retest coefficients of 0.5–0.7 over even short periods of 1 day to 2 weeks (Krueger & Schkade, 2008).

## Distinguishing Scale Norming from Adaptation and Reference Points

**Fig. 4:**
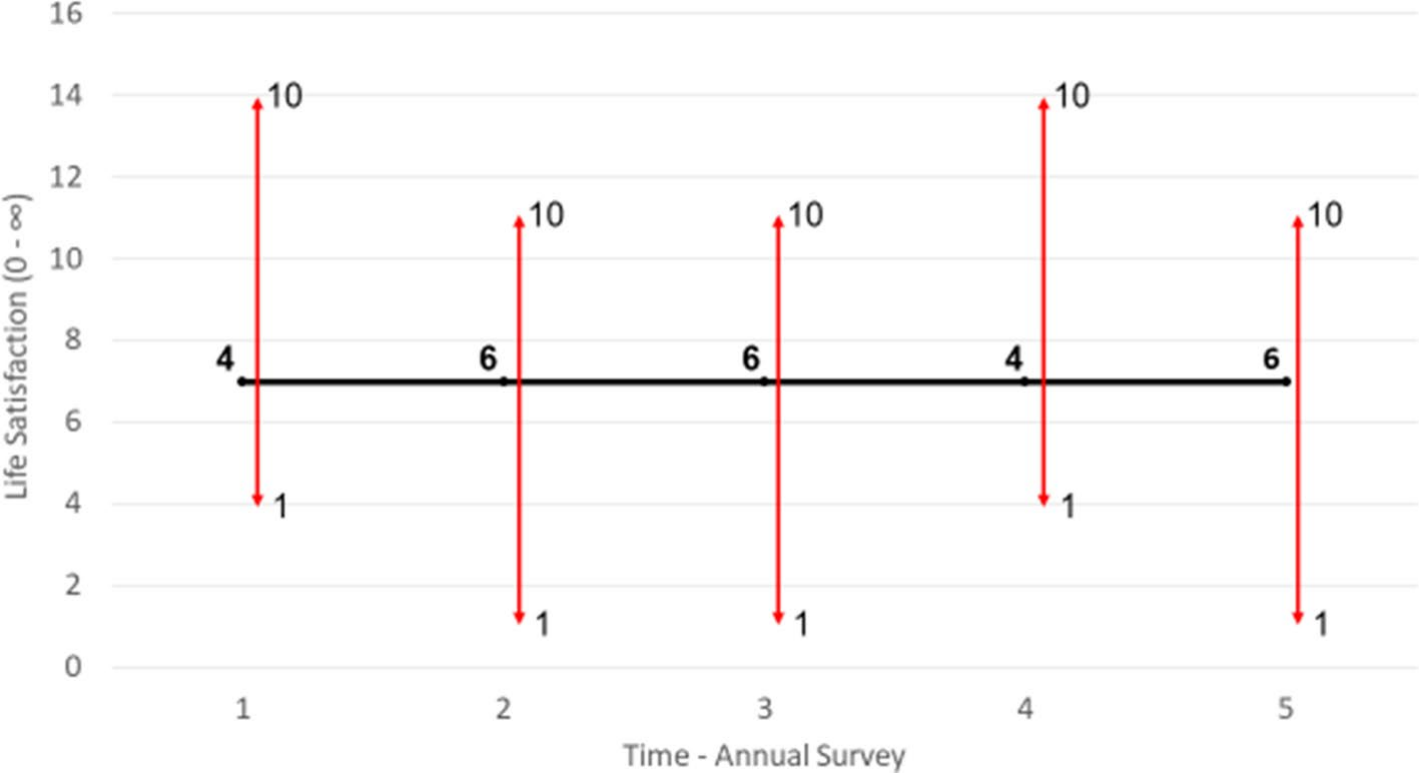
Scale norming. *Notes*: Scale norming is where the qualitative meaning of the response categories on a respondent’s scale (represented by the 1–10 vertical lines) changes over time. As a result, responses (the bold numbers) vary over time even as life satisfaction (the trend line) remains constant

**Fig. 5:**
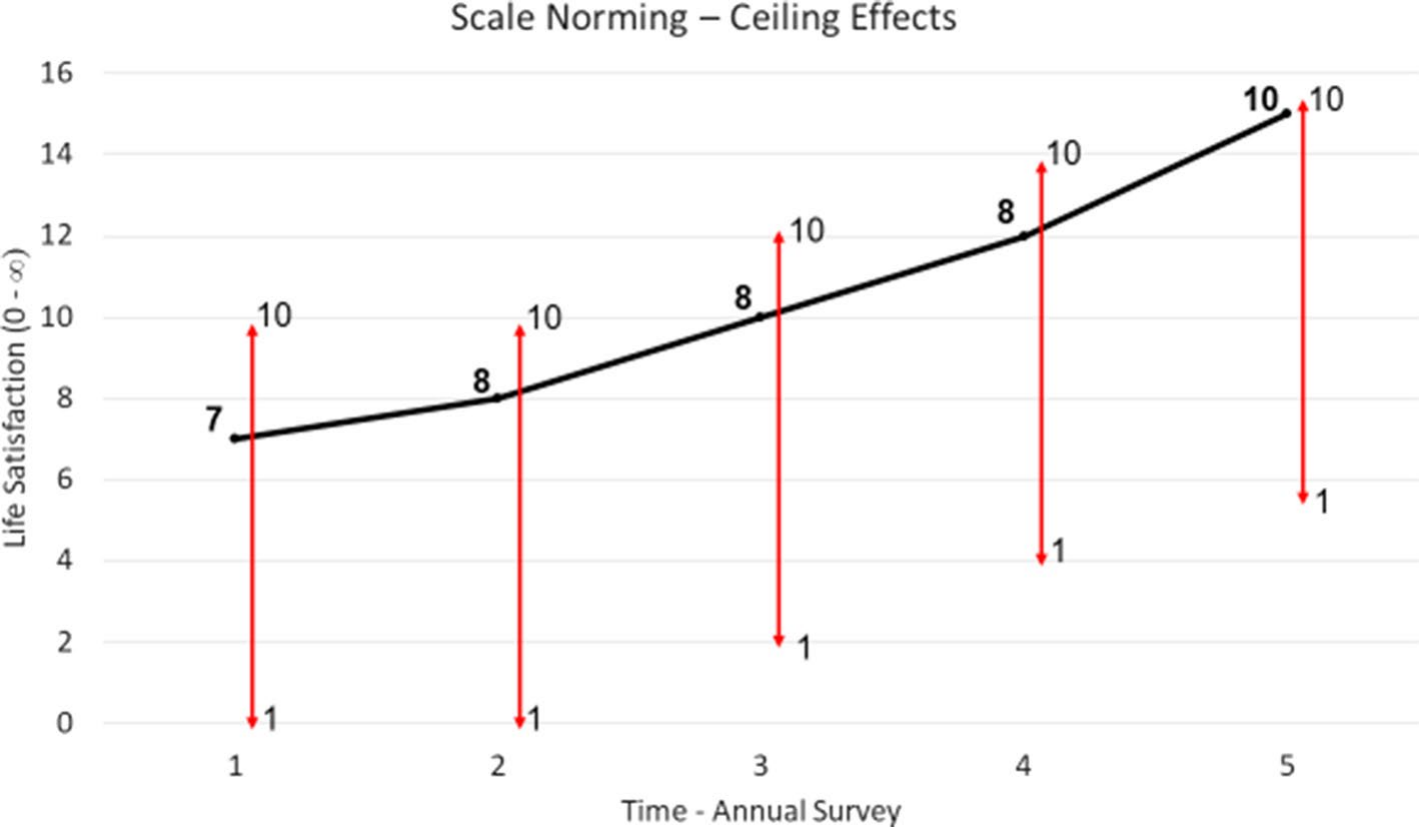
Ceiling effects. *Notes*: Ceiling effects are when a lack of space to communicate further changes at the extreme ends of a scale (here the upper end) triggers scale norming

The reporting function helps to illuminate the differences between scale norming, adaptation, and changing reference points. Scale norming is depicted graphically in Fig. 4. The y-axis tracks latent life satisfaction^1^ numerically. This is somewhat at odds with Fleurbaey and Blanchet’s model, which describes life satisfaction as a vector, that is, having only a magnitude and a direction (from low to high perhaps). A numerical scale is used here only for convenience to aid discussion—it allows for straightforward comparisons between latent life satisfaction and responses to life satisfaction scale questions. The x-axis tracks annual waves of a survey, such as HILDA or the GSOEP. In Fig. 5, the individual’s latent life satisfaction does not change across the 5 waves of the survey, staying consistently at 7. However, the scale they use does. In time 1, for example, they report 4/10, whereas in time 2 they report 6/10. Perhaps they calibrate their scale differently in these two survey instances. The result of this scale norming is apparent volatility in the respondent’s life satisfaction, but this interpretation is entirely the product of incorrectly assuming identical scales across waves.

One potential source of scale norming is ceiling effects (Wang et al., 2008). This is where an individual lacks space within the extreme regions of their scale to communicate further improvements or declines in their life satisfaction. It is depicted graphically in Fig. 5. The respondents begins in wave 1 with a high latent life satisfaction of 7, which they report directly as 7/10 on their scale. Their satisfaction continues to rise over time. At first, they can translate these improvements to their scale response, reporting 8/10 in wave 2. But then eventually they reach a point where they should report 10/10 on their original scale. This would make it impossible to communicate even more improvement in their satisfaction, which they can foresee. Additionally, in the context of Cantril’s ladder type questions, they may not want to imply that they have reached their “best possible life”. In consequence, they alter their scale rather than their response, once again reporting 8/10, except that now this corresponds to a higher level of satisfaction than the 8/10 they reported in wave 2. They continue to report 8/10 in successive waves even as their latent life satisfaction continues to rise. Researchers inattentive to scale norming might incorrectly infer that this respondent’s life satisfaction is not rising over time. Ceiling effects are consistent with results from Rasch analysis of life satisfaction scales, which find that scale use is insensitive at high response categories (Schutte et al. 2019). Ceiling effects might explain why it appears exceedingly difficult to raise average life satisfaction above 8/10 in advanced nations (Clark et al., 2018)—latent satisfaction may in fact be rising, but it is covered up by scale norming.

**Fig. 6:**
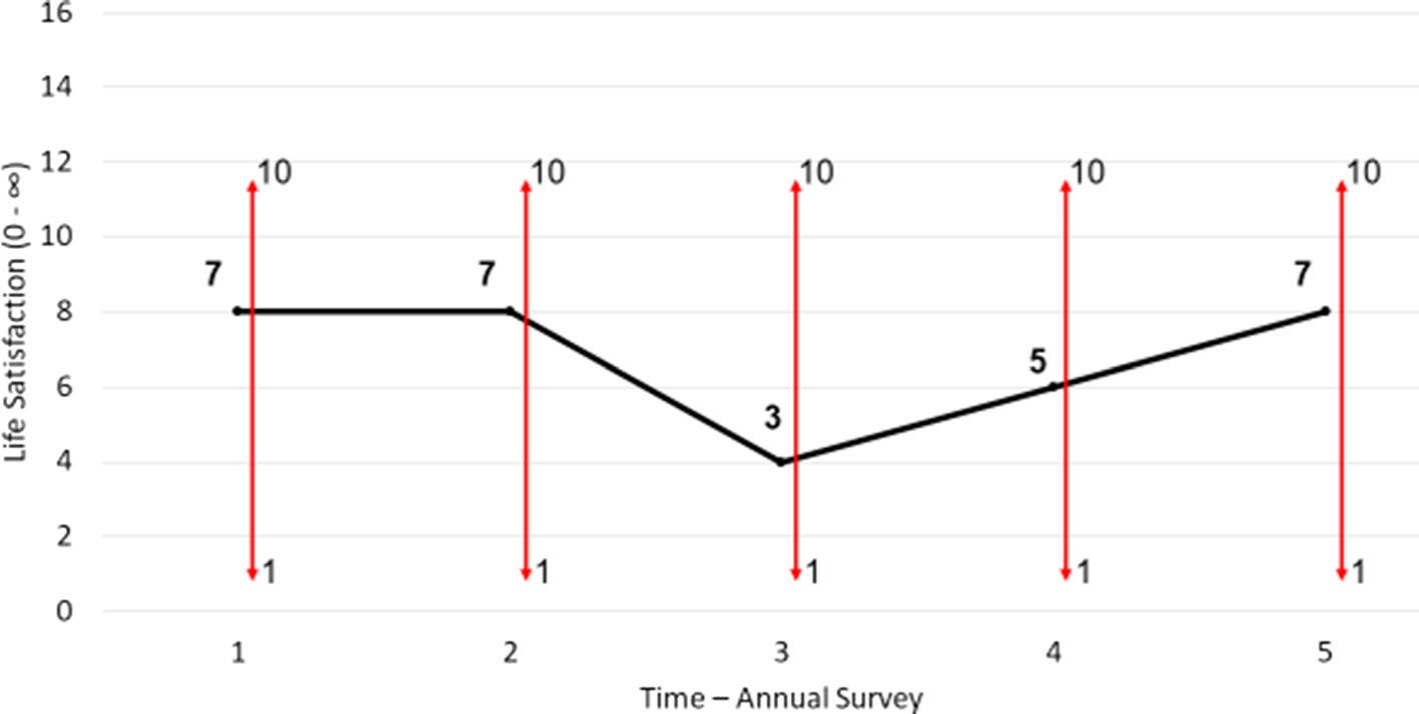
Adaptation. *Notes*: Adaptation involves real changes in latent life satisfaction rather than reporting style. Even as the scale (the horizontal bars) remains fixed over time, life satisfaction varies due to acclimatising to the shock in wave 2

Adaptation is about acclimatising to shocks that affect latent life satisfaction. In contrast to scale norming, it involves a real change in life assessment, rather than being a reporting or measurement artefact. Adaptation is depicted graphically in Fig. 6. The individual suffers a negative shock to their life satisfaction in wave 3; perhaps they burn their forearm and the skin is permanently affected with scarring. This drops their latent life satisfaction to 3 and their scale response to 3/10. Over the next two waves of the survey, they acclimatise to this shock and their life satisfaction rises commensurately. Perhaps they become accustomed to the sight of their skin and are no longer embarrassed by people’s reaction to it. In any case, by wave 5 they are back at their original satisfaction of 7 and scale response of 7/10. Importantly, no change in objective circumstances is responsible for the adaptation. The scar remains. There is also no scale norming. Adaptation is not about how one *reports* one’s life satisfaction, but about how one *feels* about things that once affected one’s life satisfaction. Adaptation and scale norming stem from different cognitive phenomena.

**Fig. 7:**
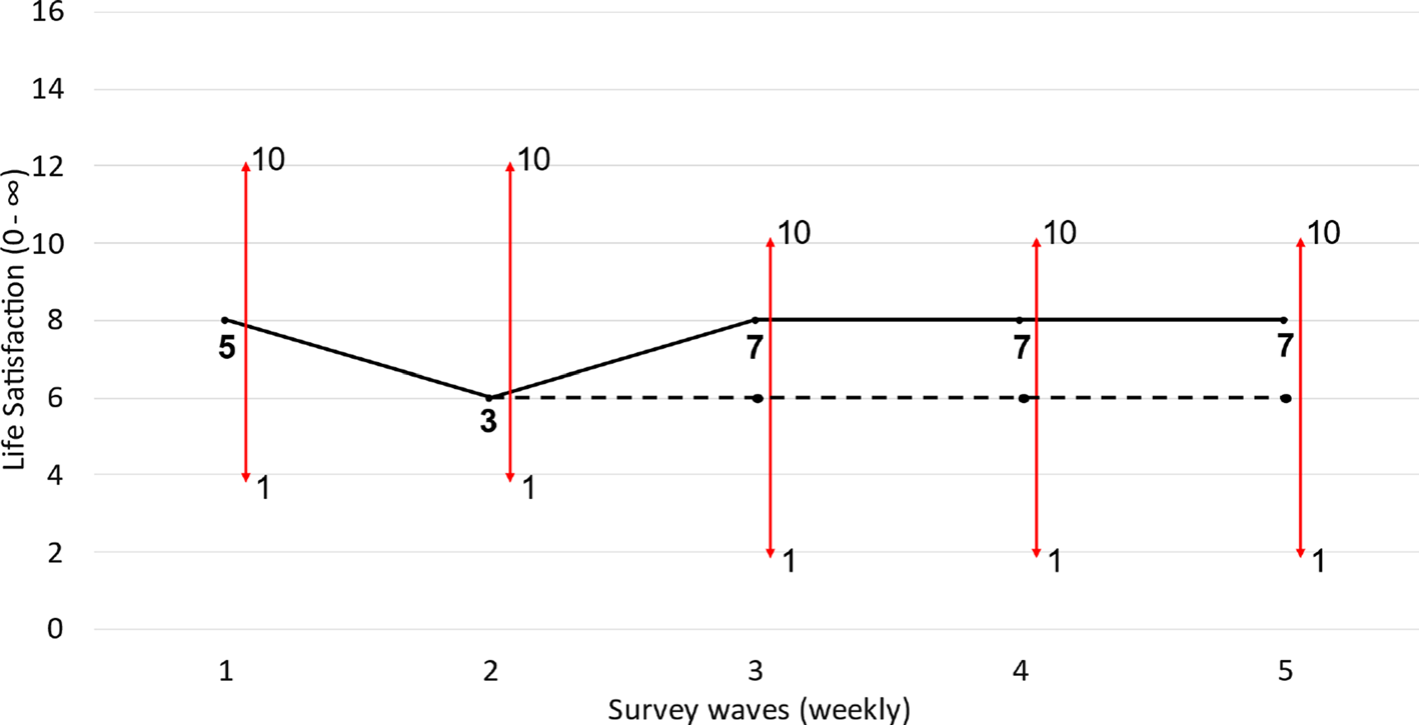
Changing reference points. *Notes*: Reference points shifts are when scale norming causes a change in feelings. This occurs between waves 2–3. The downward shift in the scale causes an improvement in latent life satisfaction. The dashed line indicates where latent life satisfaction would be had only scale norming occurred rather than a reference point shift

Life satisfaction reporting is further complicated by changing reference points. This is where scale norming causes a change in latent life satisfaction. Reference point shifts are the fundamental mechanism of many positive psychology interventions. Consider gratitude (Emmons & McCullough, 2004). This technique involves reflecting on the positive things in your life to make them more cognitively salient. For example, during COVID induced lockdowns we might focus on how grateful we are not to be ill rather than our inability to go to the gym. This deliberate, conscious effort to adjust the standard by which we assess our life causes a real improvement in feelings. This reference point shift is depicted graphically in Fig. 7. The individual initially suffers a decline in latent life satisfaction with the onset of lockdown between waves 1–2. There is no scale change. They then practice gratitude. This results in scale norming in wave 3: the scale shifts downwards, indicating a more generous reporting style. This scale norming causes a real change in feelings, with latent life satisfaction rising. The dashed line indicates where life satisfaction would have been had there been scale norming without an attendant change in feelings. The causal channel from scale norming to changes in latent satisfaction is essential to the definition of reference points shifts. An individual could scale norm while experiencing an unrelated change in life satisfaction. They could change their scope from themselves to their family while being happier about their progress in a hobby, for example. This would not be a reference point shift.

To summarise:

- *Scale norming* is where the qualitative meaning of the points on a respondent’s scale change over time. It does not involve a change in latent life satisfaction. It instead reflects a change in reporting style. More formally, scale norming involves a change in ${\xi }_{i}$ rather than a change in ${l}_{i}^{*}$ relative to ${{\varvec{l}}}_{{\varvec{i}}}$.
- *Adaptation* is where an individual acclimatises to shocks to their life satisfaction over time. This a change in latent life satisfaction (${l}_{i}^{*}$ relative to ${{\varvec{l}}}_{{\varvec{i}}})$ not a change in reporting style (${\xi }_{i})$.
- *Reference point shifts* are where scale norming *causes* a change in latent life satisfaction. The causal channel is critical: scale norming, adaptation, and exogenous shocks to life satisfaction can take place independently and simultaneously without reference point shifts occurring.

A concern for research using life satisfaction scales is that it is challenging to distinguish scale norming, adaptation, and changing reference points without information beyond scale responses. Consider the long-running study of the SWB of people with spinal injuries. While adaptation to spinal injury and other injuries is far from complete (Lucas, 2007), it does seem to occur to some extent (Hanson et al., 1993). Scale norming might also be occurring (van Leeuwen et al., 2012; Schwarz et al., 2018). People may report 7/10 before their injury and again 3 years after, but these 7 s correspond to different levels of underlying life satisfaction. Kahneman (1999) argues that the life satisfaction of people with spinal injuries could also be explained by changing reference points. Scale norming occurs, with the injured moving over time to assess their lives within the context of their injuries. This revised context helps to ease the psychological pain of the injury, and latent life satisfaction rises as a result.

A range of SW-B findings need to be regarded as open in light of the inability to separate scale-norming, adaptation, and reference point effects. International comparisons of life satisfaction, for example, may be riven with measurement error from differences in reporting style. There is some evidence for this for the sub-domain of income satisfaction from a study by Kapteyn et al. (2013). They find that after adjusting for the use of discrepant scales using vignettes, difference in income satisfaction between Americans and the Dutch in raw data disappears. This concern generalises to any intergroup comparison where the objective context of the two groups is meaningfully different, such as rural–urban or between cultures. Finally, the possibility of scale norming suggests that we should be cautious about presenting and comparing the effect sizes of various treatments on life satisfaction. Unfortunately, the accurate measurement of effect sizes is important in welfare analysis for policy. Welfare analysis requires a comparison between the latent life satisfaction, *l*_i_*, of individuals and the monotonic aggregation of such latent life satisfactions into group welfare, such as in social welfare functions (Adler, 2019). Scale norming can easily muddle this process, producing unintended consequences.

## Empirical Evidence for Scale Norming and Biased Reporting

There is some evidence for scale norming from vignette studies. Kapteyn et al. (2013) has already been mentioned. In an earlier paper, Kapteyn et al. (2010) reported similar findings where the difference in the marginal effect of income on life satisfaction between Dutch and American respondents disappeared after controlling for scale inconsistency. Angelini et al. (2013) found large differences in scale use across 10 European countries. After correcting for these using vignettes, Denmark fell from the most satisfied nation to 5th. Another vignette study by Montgomery (2017) found that correcting for discrepant scale use reduced the marginal effect of being a woman on life satisfaction from 0.09 to 0.04.

There is also a relatively large body of research on ‘response shift’ that attests to the existence of scale norming (Daltroy et al., 1999; Lacey et al., 2008; Schwarz & Sprangers, 2000). The response shift literature is concerned with understanding whether people affected by medical conditions and treatment experience changes in ‘quality of life’, measured using a variety of metrics. As in life satisfaction studies, a challenge to this research is people using different standards to evaluate their life before and after afflictions and treatment. McClimans et al. (2012) present an instructive example (p. 1862). A gentleman in the early stages of chemotherapy was asked “were you limited in pursuing your hobbies or other leisure time activities?”. He answered: “my hobby is working in the garden, that’s very difficult, quite a bit”. Four weeks later, he is asked the same question and replies: “I’m reading at the moment; gardening is not possible anymore, a little”. It is unclear to what extent the chemotherapy has affected this individual’s life. On a revealed preferences account, the man would clearly prefer to garden than read, otherwise he would have read when both options were available. However, it’s possible that having been forced to read by chemotherapy, the gentlemen discovered that he quite enjoyed reading and the activity compensated for lost quality of life from gardening. The response shift literature is interested in teasing apart changes in the man’s quality of life from measurement issues. A paper by Ubel et al. (2010) links this literature directly to scale norming. The paper argues (p. 465) that the response shift literature “lumps together sources of measurement error (e.g. scale recalibration) with true causes of changing quality of life (e.g. hedonic adaptation)”.

Schwarz et al. (2006) conducted a meta-analysis of 19 response shift studies. They found evidence for response shift (scale norming) across the studies and significant heterogeneity in the sign of the effect. Encouragingly, the size of the effect was generally small. They write that (p. 1540):

One may tend to conclude that response shifts are a common and significant phenomenon in QOL measurement, implying that people adapt their internal standards of QOL in response to changing health state. We found that overall the effect size of response shift phenomena published to date are relatively small according to Cohen’s (1992) criteria. Even a small response shift may, however, result in an underestimation of the true QOL change, i.e. concluding that it is small when it is moderate, or moderate when it is large.

While the small effect sizes Schwarz et al. observed are arguably unproblematic in the quality of life context, they are more pernicious to many policy applications of life satisfaction data. For example, cost–benefit analysis would be distorted when comparing interventions afflicted by different degrees of scale norming. Scale norming could also compound over time, especially when driven by ceiling effects, limiting the usefulness of life satisfaction scale data for longitudinal analysis. Trends in life satisfaction would give a distorted picture of social progress, for example, or long term gains from structural changes in policy settings.

One concern with the response shift literature is that it mostly relies on the ‘thentest’ methodology to determine the extent of scale norming (Schwarz & Sprangers, 2010). The thentest is any kind of retrospective pretest–posttest design such as Stillman et al.’s (2015) question to the Tongan migrants of “how did you feel when you last lived in Tonga?”. Effective implementation of the thentest requires an experimental design with treatment and control groups. A significant discrepancy between the pretest-thentest scores of the experimental and control groups suggests scale norming. This is especially the case if objective indicators of quality of life would suggest a discrepancy, as with the rising incomes of the Tongan migrants to New Zealand. However, even in such case, there are three concerns with the then-test. Schwarz and Sprangers (2010, p. 457) explain:

The first disadvantage of the thentest is that the basic premise of a shared internal standard by thentest and posttest has not been unequivocally supported (compare Sprangers et al., 1999 to Nolte et al., 2009). Second, the thentest is susceptible to recall bias, given its retrospective nature (Ahmed et al., 2005; Rapkin & Schwarz, 2004; Schwarz et al., 2004). Third, there is the potential contamination due to other alternative explanations, such as social desirability responding, and effort justification (Howard & Dailey, 1979). Further, the thentest is prone to implicit theories of change, where patients use a general heuristic for thinking about times past and infer what their initial state must have been (Norman, 2003).

Schwarz and Sprangers (2010) offer recommendations for best practice in research design when applying the thentest. These are outside the scope of discussion here, but important for analysing scale norming in life satisfaction studies going forward. What is relevant to the present paper is their concern about “potential contamination due to…social desirability responding, and effort justification…implicit theories of change”. There is a tendency among SWB scholars to use the latter two of these concerns to dismiss evidence of scale norming as a function of ‘bias’ and thereby justify the status quo. But while it is sensible to be concerned about these issues, they are arguably an inextricable part of the reporting function. Current methods of collecting life satisfaction scale data do not obviate against them, as will be explained below. As such, the only robust way forward in life satisfaction research is to take the cognitive complexity of the reporting function seriously and unpack it empirically.

*Effort justification* is where people validate sacrifices they made to realise a goal by elevating the attractiveness of that goal. For example, an ambitious lawyer might work hard to become partner at a firm but find that the job is not as good as they expected. However, they convince themselves that it is actually excellent to justify all the sunk investment they made to reach their present position. In the context of life satisfaction change, imagine a respondent who reports 8/10 in both waves 1 and 2 of a survey, while also saying that their life is better now than before (as in Köke & Perino, 2017). This could be taken as evidence of scale norming, but could also be the product of effort justification. In the quality of life studies literature, there is often some objective yardstick against which ‘better life’ can be judged. This allows researchers to reasonably claim that someone’s life is not better even if they say it is. For example, someone might say that they feel more energetic, but objectively they still can’t walk up a flight of stairs. Unfortunately, such objective indicators are fewer in the SWB space. Moreover, the whole point is to measure *subjective* well-being. This makes it more difficult (but also more imperative) to prove effort justification rather than merely invoke it.

An instructive example of *theories of change* comes from Ross (1989). He assessed people’s study skills using objective measures and self-reports. He then put students through a course designed to improve their study skills. Objectively, the course had no effect on skills. Student self-reports seemed to parallel this, with students who were objectively 6/10 also assessing themselves as 6/10. However, at the end of the course, Ross had students recall their study skills from the start of the course. Students who reported themselves as 6/10 at the end of the course would remember themselves as 4/10 from the start of the course. They downgraded their earlier assessment because of an implicit theory that the course would improve their study skills. Ross’ experiment shows how theories of change could explain the apparent scale norming of the Tongan migrants in Stillman et al.’s (2015) study.

Ross’ (ibid.) experiment makes clear how theories of change can be a concern in SWB assessment, but its experimental context is significantly different from assessment of life satisfaction. Multiple lines of inquiry into subjective well-being give a central and positive role to theories of change. Narrative therapy works by helping patients order their lives according to a theory of change (Angus & McLeod, 2004). Eudaimonic theories of well-being emphasise self-actualisation (Fabian, 2020). This involves positing some ideal or true self that one is progressing towards over time, which is necessitates a theory of change. Goal setting and achievement, which is widely implicated in subjective well-being, is similarly impossible without some theory of change. More broadly, theories of change are likely to inform the reporting process. The ranking problem, for example, might involve ordering a possible life, *l*_*a*_, higher than another, *l*_*b*_, because inherent in *l*_*a*_ is greater progress towards some desired outcome. The calibration problem could be influenced by how an individual breaks down incremental steps in an implicit theory of change. For example, they may associate a 6/10 with graduating, a 7/10 with getting married, an 8/10 with buying a house, and a 9/10 with having kids. Constructing a standard and assessing one’s life against it is likely to involve an implicit theory of change because life is a dynamic, intentional, process. We therefore need to conceptually differentiate between *erroneous* theories of change, depicted in Ross’ (ibid.) study, and germane theories of change that provide a framework for SWB evaluations.

The discussion above addressed the complexity of invoking effort justification and implicit theories of change as explanations for trends in life satisfaction scale data that might otherwise indicate scale norming, such as the observations in GSOEP and BHPS discussed in the introduction to this paper. This leaves issues of recall bias and errors of memory unaddressed. To this end, the second half of this paper analyses an experiment that explores scale norming in such a way that its results are hard to dismiss as merely the product of recall bias. The purpose of this experiment is to contribute to the empirical evidence that scale norming is a real phenomenon in life satisfaction scale data, not merely the product of cognitive biases. The experiment is not a test or illustration of the conceptual framework outlined in parts 1 and 2 of the paper. That would require a much more sophisticated and resource-intensive survey.

## An experiment to assess scale norming as the product of recall bias

**Fig. 8:**
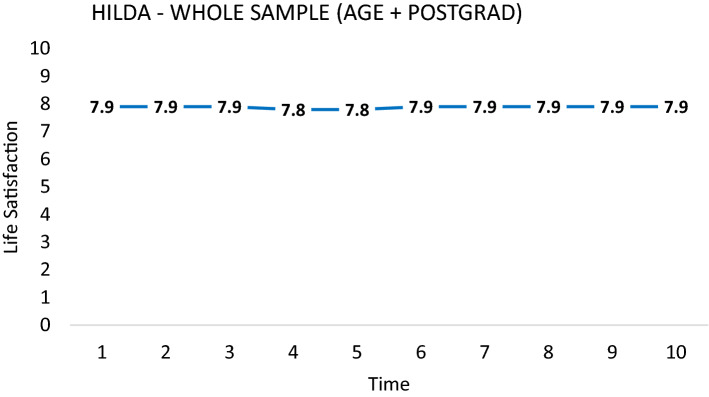
Average life satisfaction of postgraduates aged 17–35 in HILDA, 2001–2010

The study of life satisfaction over time typically involves plotting year-on-year responses to life satisfaction scale responses into a time series. Figure 8 provides an illustrative example from the first 10 waves (2001–2010) of the Household Income and Labour Dynamics of Australia (HILDA) panel. The x-axis tracks the waves of the survey, while the y-axis shows average life satisfaction within the sample measured using the following life satisfaction scale question: “All things considered, how satisfied are you with your life at this time on a scale of 1–10?”.

**Fig. 9:**
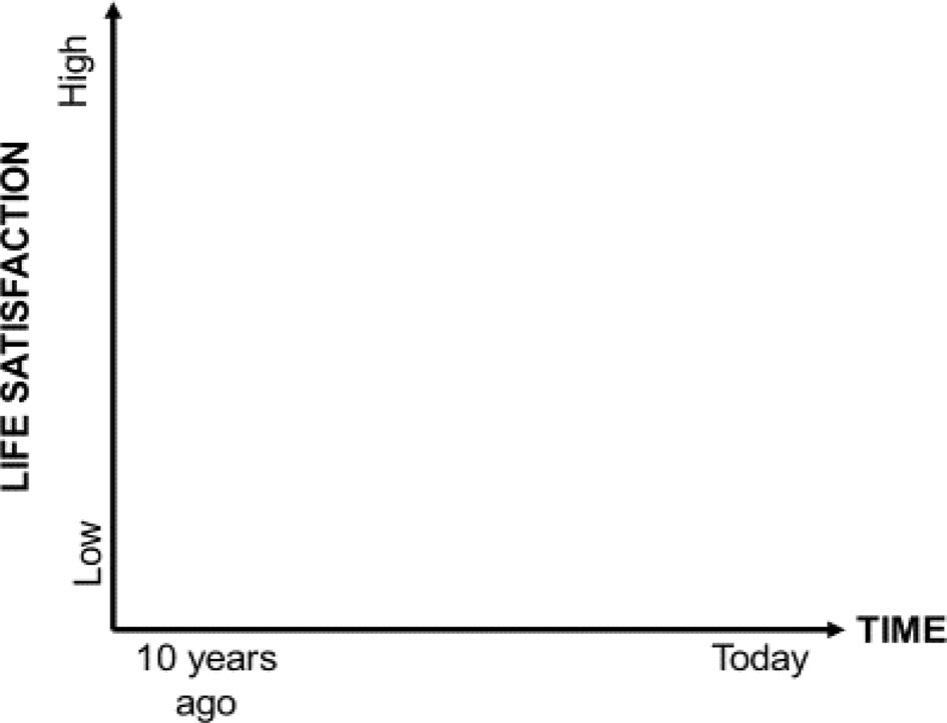
Life satisfaction plot. *Notes*: sample restricted to individuals aged 17–35 who obtain a postgraduate qualification by wave 10

If scale norming occurs over time, we might expect to see a different shape in the time series of life satisfaction if we ask respondents to *assess an entire period using a single scale*. This intuition was used to inform a simple survey question, the *life satisfaction plot*, depicted in Fig. 9. As in Fig. 8, the x-axis tracks time while the y-axis tracks life satisfaction. Respondents are presented with this graph and asked the following:

**Question 1:** In the area below, please draw a **line graph** depicting your life satisfaction levels over the past 10 years:

Respondents were also asked the life satisfaction scale question from HILDA:

**Question 2:** All things considered, how satisfied are you with your life at this time on a scale of 1–10?

As well as the following retrospective question:

**Question 3:** Think back to 10 years ago. Meditate for a moment on the activities you did, the concerns you had, the good and bad things that were in your life and how happy or sad you were. Place yourself back in your mind at the time.

*10 years ago*, what would you have answered to the following question: all things considered, how satisfied are you with your life on a scale of 1–10?

Scale-norming can be investigated by comparing trends in life satisfaction plots with responses to the two scale questions. If respondents show significant change in one direction in their life satisfaction plots but no commensurate change (or an inverse change) in their scale response from t_1_ to t_10_ this would suggest (though not prove definitively) that they are scale-norming. A high scale response score for t_1_ coupled with a sustained upward trend in their plot would add further credence to this inference as this might indicate ceiling effects kicking in. Adaptation can be studied by looking for convergence back to a baseline following shocks. The 10-year time horizon provides enough scope for this to occur.

### Data and Methods

The three-part survey was administered to two samples. The first consisted of 278 masters students at a top 100 research intensive university. The survey was administered by hand in this instance. A transparency with a 10 × 10 grid on it was overlaid onto the plots drawn by respondents to derive life satisfaction scores from 1 to 10 (in increments of 0.5) for years 1–10. This process quantifies the shape of the plots. The second sample consisted of 1050 Australians representative by age, gender, and location (see “Appendix 1” for summary statistics of demographic variables). In this case, the survey was administered online with the help of IPSOS/i-view, a reputable polling and market research company. The respondents drew their plots using a 10 × 10 grid of boxes that they could click across to create a line graph, with the software connecting the dots for them. The survey questions were permuted in both samples; by class in the student sample (6 groups) and at the individual level in the online sample. This permutation had no noteworthy effects on responding (see “Appendix 2” for a full analysis).

Three further variables necessary to investigate scale norming are calculated from responses to the three survey questions. These are:

- The net change in respondents’ life satisfaction from t_1_ to t_10_ as depicted in their *plots,* i.e. question 3. This variable is defined as^2^:
  $$net change=\left({t}_{2}-{t}_{1}\right)+\left({t}_{3}-{t}_{2}\right)+\left({t}_{4}-{t}_{3}\right)+\left({t}_{5}-{t}_{4}\right)+\left({t}_{6}-{t}_{5}\right)+\left({t}_{7}-{t}_{6}\right)+\left({t}_{8}-{t}_{7}\right)+\left({t}_{9}-{t}_{8}\right)+\left({t}_{10}-{t}_{9}\right)$$
- The total volume of absolute change, positive or negative, in their *plots,* defined as:
  $$total change= \left|{t}_{2}-{t}_{1}\right|+\left|{t}_{3}-{t}_{2}\right|+\left|{t}_{4}-{t}_{3}\right|+\left|{t}_{5}-{t}_{4}\right|+\left|{t}_{6}-{t}_{5}\right|+\left|{t}_{7}-{t}_{6}\right|+\left|{t}_{8}-{t}_{7}\right|+\left|{t}_{9}-{t}_{8}\right|+\left|{t}_{10}-{t}_{9}\right|$$
- The change in their life satisfaction as reported in their responses to the *scale questions,* i.e. questions 1 and 2, defined as:
  $$change={Life Satisfaction}_{now}-{Life Satisfaction}_{then}$$

Where “now” corresponds to the life satisfaction scale question about present satisfaction, and “then” corresponds to the question about satisfaction ten years ago.

## Results

**Fig. 10:**
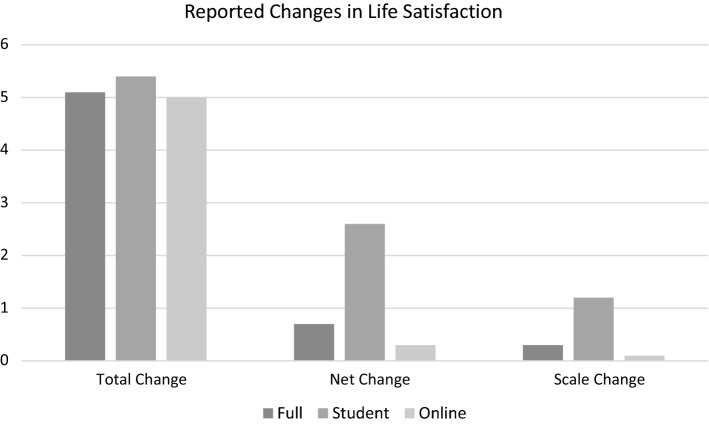
Reported changes in life satisfaction across plots and scales by sample

**Fig. 11:**
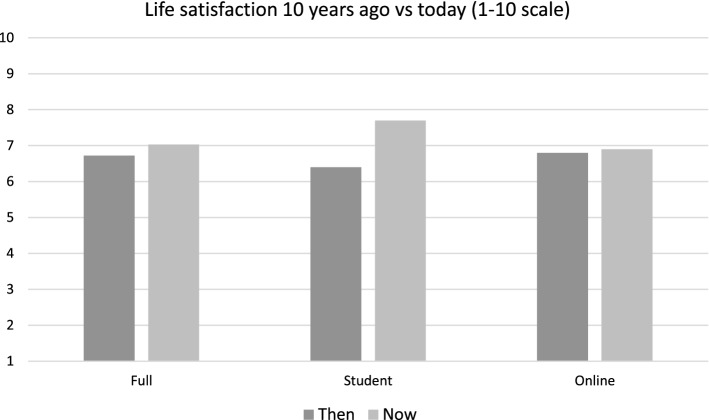
Life Satisfaction “then” versus life satisfaction “now”

**Fig. 12:**
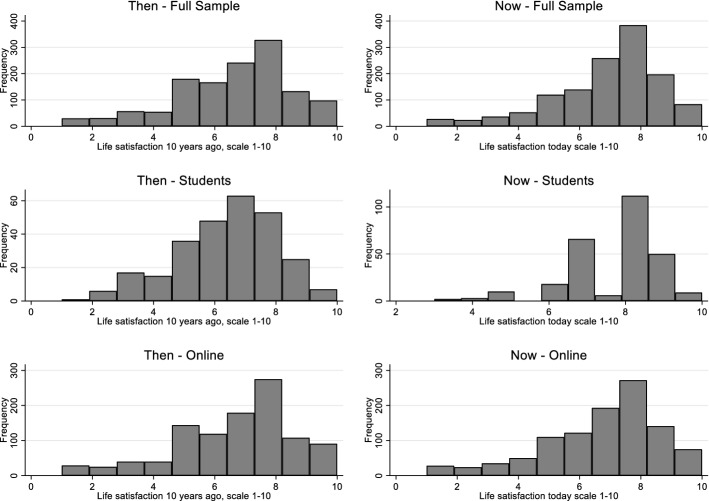
Distribution of life satisfaction scale responses by sample

**Fig. 13:**
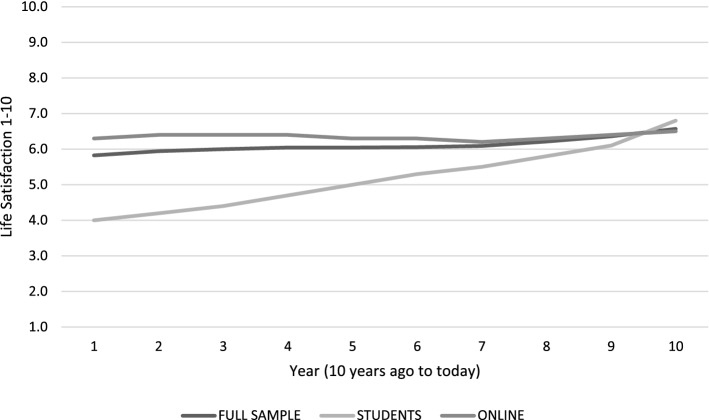
Average trends in plotted life satisfaction by sample

Table 1 and Figs. 10, 11, 12 and 13 provide summary statistics broken out by sample. The student sample experienced markedly more appreciation in their life satisfaction, with an average net change (plotted) of 2.6 and an average change (in scale response) of 1.2 compared to an average net change for the online sample of only 0.3 and average change of only 0.1. Both samples show similar volumes of total change with an average of 5.4 among the students and 5 for the online sample.

**Table 1:** Summary statistics for key variables by sample

	Full sample		Students		Online	
Variable	Mean	S.D	Mean	S.D	MEAN	S.D
Total change	5.1	4.6	5.4	3.8	5.0	4.8
Net change	0.7	3.5	2.6	3.0	0.3	3.4
Change	0.3	2.4	1.2	2.0	0.1	2.5
LSAT “Now”	7.0	2.0	7.7	1.2	6.9	2.1
LSAT “Then”	6.7	2.1	6.4	1.8	6.8	2.1
Obs	1328		278		1050

### Scale-norming analysis

**Fig. 14:**
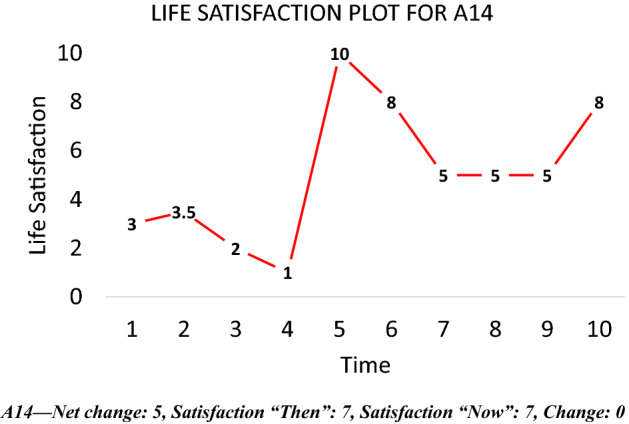
Example of scale-norming from plotting experiment

A respondent with strong movement in one direction in their plot who does not show any commensurate change in their scale response may be scale-norming over time. A graphical example drawn from the experimental data is depicted in Fig. 14. The individual has a discrepancy in their plot of + 5 but their scale response doesn’t change at all from ten years ago to today. This makes little sense unless the individual’s scale changes qualitatively over that period.

This phenomenon can be explored by adopting a loose definition of scale-norming designed to catch any potential candidates and then gradually tightening the definition to weed out reasonable respondents. This honest approach makes clear how tight the definition needs to be before the phenomenon disappears. Scale-norming is initially defined as having an absolute value of net change of greater than or equal to one standard deviation but an absolute value of scale change of less than or equal to 1 i.e. the smallest amount of non-zero scale change possible. Intuitively, what this definition does is ask whether someone has had meaningful movement in their life satisfaction as plotted but this movement has not shown up proportionately in their scale responses.

The loose definition returns 157 candidates, or 11.8% of the full sample. However, many of these are making arguably reasonable responses, such as a net change of 3.5 for a change of 1. Perhaps this is simply how much life improvement is required to generate an increase in the bounded scale, in which ‘space’ is scarcer than in the plot. The definition is therefore tightened in the next step of the analysis: the threshold for net change is retained at one standard deviation, but the threshold for change is reduced to less than 1 (i.e. no movement). This leaves only 71 candidates, or 5.3% of the sample. Some of these quite clearly show scale-norming, such as one individual reporting a net change of 9 and a scale change of 0. Indeed, there are 20 respondents (1.5%) showing an absolute net change of more than 2 standard deviations (i.e. 7 +) but no scale change whatsoever.

**Fig. 15:**
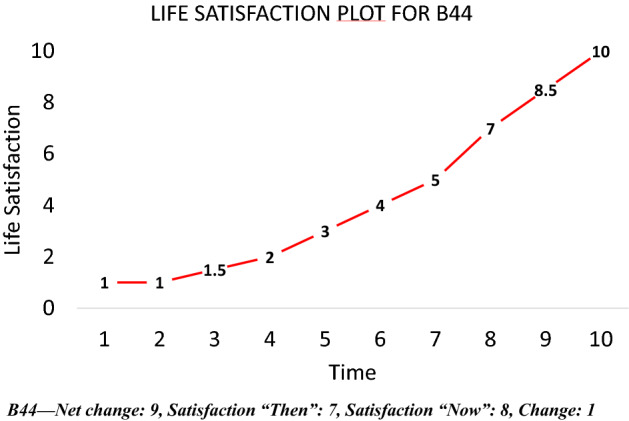
Example of scale-norming by wide definition

In a final step, a strict but *wide* definition of normalisation is used where respondents are considered to have normalised if they report 2 standard deviations of net change or more (i.e. 7 +) but no more than 1 unit of scale response change. Such individuals show a large change in their life satisfaction in their plots but not a *proportionate* change in their scale response. A graphical example of such a respondent is presented in Fig. 15. This individual has a netchange of + 9 but a scale change of only + 1. It is debatable whether these individuals are scale-norming. In any case, there are only 11 respondents of this sort who are not counted in the second phase of tightening. Adding these two groups together gives 82 candidates, or 6.2 per cent of the sample. The total number of candidates rises inconsequently to 6.4% if the full sample is broken out into the student and online sub samples as they have divergent standard deviations for net change. These encouragingly low numbers are summarised in Table 2.

**Table 2:** Number of unique respondents potentially scale norming by sample

Scale-norming definition	Full sample	Students	Online
(Weak) Net change > = 1 S.D. |change|< = 1	157 (11.8%)	53 (19.1%)	114 (10.9%)
(Medium) Net change > = 1 S.D. |change|< 1	71 (5.3%)	16 (5.8%)	57 (5.4%)
(Strong) Net change > = 2 S.D. |change|< 1	20 (1.5%)	6 (2.2%)	18 (1.7%)
(Wide) Net change > = 2 S.D. |change|< = 1	31 (2.3%)	11 (4%)	25 (2.4%)
[TOTAL] Unique (Medium) + (Wide)	82 (6.2%)	21 (7.6%)	64 (6.1%)

Number in brackets is the number of candidates as a percentage of sample in question. The final row sums candidates from the medium definition and additional unique candidates from the wide definition to give a (debatable) total number of individuals scale-norming

**Fig. 16:**
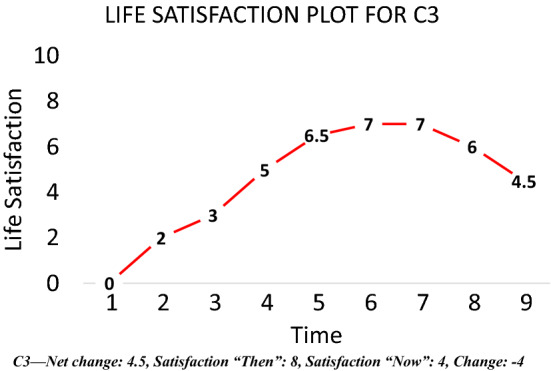
Example of a reverser

There are additional candidates for scale-norming who have not yet been considered—*reversers*. A reverser is someone whose scale response moves in the opposite direction to their plot, such as the respondent in Fig. 16. They plot an increase of life satisfaction of 4.5 but their scale response declines by −4. This makes little sense unless the individual is scale-norming over time. There are 103 reversers in the full sample. A more detailed breakdown is provided in Table 3. 98 of these reversers do not appear in either the medium or wide definition of scale norming. Adding them to the 82 unique scale norming candidates from the full sample yields 180 individuals whose responding is consistent with scale norming. This is 13.6% of the sample, a less encouraging number.

**Table 3:** Number of unique reversers by sample

Reversal definition	Full sample	Students	Online
Netchange > 0 but change < 0	52 (3.9%)	17 (6.1%)	35 (3.3%)
Netchange < 0 but change > 0	51 (3.8%)	9 (3.2%)	42 (4%)
Total	103 (7.8%)	26 (9.3%)	77 (7.3%)

Number in brackets is the number of candidates as a percentage of sample in question

### Adaptation analysis

**Fig. 17:**
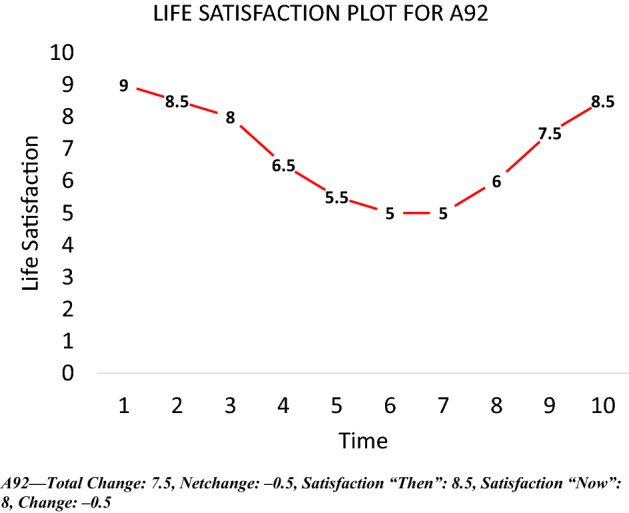
Example of adaptation

The conceptual analysis above implied that the strength and speed of adaptation observed in longitudinal life satisfaction studies (Sheldon & Lucas, 2014) may be overestimated due to an inability to account for scale norming. To investigate the severity of this concern, the section below explores adaptation dynamics in the plot data. The investigation begins as before with a weak definition that should capture all relevant respondents. This is then tightened. The weak definition is total change (in plot) of more than 1, but less than or equal to 1 net change (in plot) and less than one absolute value of change (in scale). Intuitively, this should capture respondents who have experienced shock(s) to their life satisfaction (total change) but then adapted back to their set point over time (hence limited net change and change). An example respondent is depicted below (Fig. 17). They experience a substantial deterioration in their life satisfaction over the 10 years of the plot, but almost entirely adapt back to their original level by the end of the period.

This very weak definition of adaptation picks up only 147 possible adapters out of 1127 individuals who have experienced the requisite total change. If the required amount of total change is increased to 1 standard deviation, the number of candidates halves to 75 out of 640 respondents with the requisite shocks. These aren’t large shocks to adapt to, and yet we see few individuals adapting to them in the manner typically identified by longitudinal studies using life satisfaction scales. These definitions might be discounting people who experience a large shock but do not have time in the 10 year stretch to adapt all the way to baseline. A wide definition is employed to examine this possibility. It is specified as total change of two standard deviations or more but a net change of no more than 1 standard deviation and a (scale) change of no more than 1. This yields 86 candidates out of 276 who have experienced sufficient total change. 49 of these candidates are unique and do not appear in earlier definitions of adaptation. This rate of adaptation is slower than what appears in longitudinal studies using scales. A summary of these results is provided in Table 4, along with breakouts by sub sample. Interestingly, a larger portion of respondents adapt back, albeit only partially, to the large shocks than the smaller ones. Perhaps our perception of adaptation as a strong force is driven in part by our study of dramatic events like divorce and spinal injury? Adaptation may be rarer or weaker following relatively minor shocks.

**Table 4:** Adaptation results

Adaptation definition	1	2	3 (%)	4 (%)
Weak (full sample)	147	1127	13	11
Tight (full sample)	75	640	11.7	5.6
Wide (full sample)	86	276	31.2	6.5
Weak (students)	11	267	4.1	4
Tight (students)	7	176	4	2.5
Wide (students)	7	54	13	2.5
Weak (online)	136	860	15.8	13
Tight (online)	69	507	13.6	6.6
Wide (online)	42	98	43	4

Weak: Total change > 1, Net change <  = 1, (scale) Change < 1

Tight: Total change > 1 S.D., Net change <  = 1, (scale) Change < 1

Wide: Total change >  = 2 S.D., Net change <  = 1 S.D., (scale) Change < 1

Column 1 is the total number of candidates meeting the criteria

Column 2 is the number of individuals in the full sample with sufficient total change to meet the criteria

Column 3 is the number of candidates as a percentage of those with sufficient total change

Column 4 is the number of candidates as a percentage of the full sample

## Discussion

Overall, the plot instrument seems to produce different time series of life satisfaction compared to longitudinal scale studies. In particular, adaptation is rare in the plots, occurring in about 11% of people experiencing a shock in the full sample (rows 3 and 4 of column 4 in Table 4). This suggests that we should explore alternate ways of measuring of life satisfaction besides scales to discern which empirical regularities are shared across empirical instruments. If we see different patterns in the data depending on which measurement instrument we are using, then we need to justify why one instrument is more trustworthy or accurate than others. Scale norming is roughly as prevalent as adaptation, occurring in 13.6% of the full sample. Research on adaptation to date has rarely taken seriously the possibility of scale norming as an alternate explanation of their results (Odermatt & Stutzer, 2019 is a rare exception). It would be appropriate for future studies to investigate scale norming where possible, or at least acknowledge it as an alternate explanation.

Given that the plot metric involves 10 years of recall, some might argue that the data it produces is riddled with recall bias. This may well be the case, but as the plot question is asked alongside the life satisfaction 10 years ago on a scale question, recall bias should affect both responses *identically*. As such, recall bias does not explain the *discrepancy* between life satisfaction as reported on the plots versus the scale questions. Yet, crucially, it is the discrepancy that implies scale norming over time.

Some readers might also be concerned that the data do not definitively prove the existence of scale norming. Notably, they could also be explained by reference point shifts. There are two rejoinders to make here. First, the purpose of this study was not to prove the existence of scale norming, but rather to show that even when recall bias is somewhat controlled for, the same patterns consistent with scale norming that we see in longitudinal panels like the GSOEP still emerge. Therefore, we should not assume that recall bias is sufficient explanation for these patterns. Second, as noted earlier, reference point shifts *involve scale norming.* What differentiates reference point shifts is that this scale norming provokes a real change in feelings. If scale norming exists in any form then we need to develop ways to account for it in welfare analysis or risk biasing our results.

The two samples possess properties that make the results robust to several other concerns. First, the three questions were delivered to online respondents amidst 57 other questions,^3^ and they were unable to go back and check what they had answered to previous questions. This could be expected to reduce respondents’ ability to make sure that they had answered the questions in a consistent manner, thereby giving a more honest picture, but may also lead to more errors of memory. Conversely, the student sample provides a tighter test of recall bias in responding as the three questions are administered one after the other. However, the students can easily check that their answers are logically consistent, so their sample is likely to understate the prevalence of scale norming. This is worth noting as results consistent with scale norming are already more prevalent among the students compared to the online sample (7.6% versus 6.1%).

Second, the y-axis in the plot metric was labelled from ‘low’ to ‘high’ rather than 1–10 to place an emphasis on the shape and trajectory of life satisfaction over time and move away from scale reporting. To assess whether this had any effect on responding, a random sub-sample of 200 online respondents was given a plot with the y-axis labelled from 1 to 10. This had no statistically significant impact on their reporting (see “Appendix 3”).

Finally, some might perceive the plotting questions as complex and thus hard to answer accurately. To investigate this, all online responses were covertly timed. The median time to answer the plot is 61 s. This shows both that respondents took the time to think through their answer and that it was not too challenging. 61 s is longer than the median times taken to answer the life satisfaction ‘now’ and ‘then’ scale questions, which are 11 and 24 s respectively (such quick times are common in this research). However, it is less time than answering 10 consecutive scale questions (i.e. 110–240 s), which suggests that respondents do not find the plot more cognitively challenging to answer than a scale question, merely more time consuming.

While the study is robust to some concerns it certainly has significant limitations. The most important is that it cannot definitively demonstrate the existence of scale norming because it does not involve data on the qualitative meaning of the points on respondent’s scales. While the trends discussed are certainly consistent with scale norming, they could also be driven by very wide intervals between points on a respondent’s scale, such that a very large net change is not enough to provoke a scale change. Reversers could be explained by careless responding, though their numerousness makes this unlikely. Future longitudinal studies should consider asking respondent’s to describe their scales verbally, such as in one tweet (280 characters) per response category. These descriptions could then be compared across time to assess similarity. Respondents could be asked directly whether they think their scale today as they have described it is meaningfully similar to their scale from prior survey waves. Higher frequency longitudinal designs could also reduce the reliance on recall of the present study. For example, following Köke and Perino (2017), a study could ask respondents *monthly* whether they are more, less, or as satisfied with their life as in the previous month. This is a short recall window. These responses could be used to generate the kind of time series plots used in the methodology of this paper, but with much less reason to fear recall bias. This plot could then be compared to annual life satisfaction scale responses to assess scale norming.

A second, related limitation is that the study provides little insight into the nature of the reporting function and cannot offer guidance on how to control for scale norming. Unfortunately, unpacking the reporting function rigorously is extremely challenging and will likely require substantial methodological innovation, so offering practical guidance at this stage is impossible. One potential approach is the use of cognitive interviewing. This involves having respondents “think out loud” when answering psychometric questions. The conceptual framework presented herein could be used to inform semi-structured questions for such cognitive interviewing. For example, after answering a life satisfaction scale question, interviewees could be asked whether they thought about their families and society when answering the question or just themselves. This could shed some light on the scope problem. If such cognitive interviewing where applied as part of evaluations of wellbeing interventions, such as gratitude studies, it could potentially illuminate the differences between exogenous changes in life satisfaction due to external shocks like COVID-19, endogenous changes through deliberate reference point shifts, adaptation, and scale norming. Another option is the to use vignettes or recalled life satisfaction to anchor longitudinal life satisfaction responses and estimate potential scale norming, as described in Kaiser (2020). This methodology has substantial limitations but can at least allow for an analysis of the sensitivity of results to scale norming. Developing methods to identify, assess, and control for scale norming in empirical work should be a high priority for life satisfaction research going forward.

Aside from scale norming evidence being robust to recall bias, perhaps the main takeaway from the experiment is that people can experience sustained trends in their life satisfaction over time without this showing up in their scale responses. This cautions against the use of longitudinal trends in life satisfaction scale responses to track social progress, as real change may be obfuscated by scale norming over time. The discrepancy between the shape of plots and time trends in scale responses also suggests that we should be cautious about developing theories of adaptation in life satisfaction from life satisfaction scale data alone, as scales make it easy for researchers to confuse adaptation and scale norming.

## Conclusions

The notion of scale norming has been around since the early days of hedonic psychology (Frederick & Loewenstein, 1999) but has received limited investigation compared to related phenomena like adaptation. Quality of life studies have examined response shift relatively extensively, but limited work has been done to port concepts, methods, and results over from that literature to life satisfaction scale scholarship. Now is an opportune time to do this for two reasons. First, advocacy is growing for greater use of life satisfaction data in economics and public policy where the scale norming is potentially highly pernicious. And second, the field of subjective well-being has attained sufficient latitude in recent decades to undertake an excavation of its methodological foundations without undermining its credibility.

This paper made several contributions to this project. First, it further developed a formal model of the reporting function that illuminates the distinction between adaptation, scale norming, and reference point shifts in the context of life satisfaction scale questions. Second, it used this model to explain why effort justification and implicit theories of change are fundamental to life satisfaction reporting and are thus not good alternate explanations for data that might indicate scale norming over time. Third, it presented results from an experiment that suggest that when recall bias and errors of memory are at least partially controlled for, scale norming still shows up. The paper thus addresses the three main ‘cognitive biases’ used to hold off concerns regarding life satisfaction scale research related to scale norming. This is a call to action: the field needs to take the cognitive complexity of answering life satisfaction scales more seriously, unpack the reporting function empirically, and ascertain the extent and severity of scale norming.

This is not to say that you cannot use life satisfaction scales for many applications in psychology, social science, and even policy. Scales at least remain psychometrically valid. However, we must be careful about aggregating effect sizes derived from scale data, especially in longitudinal research. Unfortunately, it is precisely aggregation that we need for cost–benefit analysis, and longitudinal research that we need for measuring social progress. Alternate indicators of well-being used in such policy applications have their own measurement problems of course, so this word of caution applies to everyone invested in this debate.

## Appendix 1: Summary statistics of demographic variables

Besides the 3 survey questions, no other data was collected from the student sample. Summary statistics reported here are *only for the online sample*, N = 1050 (Table 5).

**Table 5:** Summary statistics of demographic variables for online sample

Variable	%	Variable	%
Age: 18–25	11.9	Employed full time	31.5
Age: 26–35	17.9	Employed part time	18.3
Age: 36–45	17.3	Not employed	52.6
Age: 46–55	12.3	Professional/Managerial	19
Age: 56–65	1.7	Unskilled/labour	4.5
Age: 65 +	38.9	Sales/clerical	10.6
Male	48.9	Technical/skilled	7.8
Female	51.1	Other occupations	7.4
High school drop out	11.9	Income: < 15,000	4.5
Finished High school	17.3	Income: 15 000–24 999	9.2
Skilled vocational	16.7	Income: 25 000–34 999	10.5
Associate diploma	10.2	Income: 35 000–49 999	28.7
Bachelor’s	20.6	Income: 50 000–74 999	15.6
Some postgraduate	15	Income: 75 000–99 999	12.1
Married	47.7	Income: 100 000–149,999	15.6
Living with partner	13	Income: 150 000–199,999	6.7
Divorced/separated	10.6	Income: 200 000 +	2.9
Widowed	5.7	Income: refused to disclose	9.9
Single/never married	22.1		
More than 5 km from town	2.7		
Rural town	16.4		
Major regional city	25.1		
Capital city	55.8		

Incomes are in Australian dollars, worth roughly 0.6 USD

## Appendix 2: Permutation of Questions

Splitting the full sample into 6 groups according to the order in which they received the survey questions turns up only one statistically significant effect associated with question order (see Tables 6, 7, 8, 9, 10, 11, and 12). Respondents in the order “then, plot, now” seem to report less total change in life satisfaction that respondents in other groups. There are no differences on other variables, and the absolute magnitude of this effect is at most 1.1 points (compared to the “now, plot, then” order—see Table 5). It is unclear from these results what might be driving this result.

**Table 6:** Summary statistics (full sample)

Question order	now, plot, then	Now, then, plot	plot, now, then	plot, then, now	then, now, plot	then, plot, now
LSATT	6.725225	6.7	6.903922	6.411638	6.8	6.752792
	(2.096904)	(1.929603)	(2.07857)	(2.208248)	(2.024738)	(2.179743)
LS NOW	7.024554	6.840541	7.188672	6.94181	7.00431	7.180203
	(1.819322)	(1.803598)	(1.964166)	(2.286289)	(1.980419)	(1.956612)
TOTAL ∆	5.40625	5.255676	5.603113	4.787554	5.081897	4.307107
	(4.218277)	(4.988953)	(6.671715)	(3.65231)	(3.71521)	(3.190124)
NET ∆	0.8392857	0.3972973	1.003891	0.77897	0.8706897	0.393401
	(3.527098)	(3.111644)	(3.679279)	(3.584489)	(3.545084)	(3.366826)
SCALE ∆	0.3040541	0.1405405	0.2854902	0.5301724	0.1934783	0.4274112
	(2.357453)	(2.280406)	(2.591519)	(2.55823)	(2.505139)	(2.296906)
N =	222	185	255	232	230	197

Means, with standard deviation in parentheses. The difference in group size is driven by differences in the sizes of the student classes. The high variance of total change in the “plot, now, then” group appears to be driven by a single outlier in the student sample who reported manic depressive trends in their plot

**Table 7:** *t* tests for statistically significant differences between question order #1 (**now, plot, then**) and other question orders

NPT	NTP	PNT	PTN	TNP	TPN
LSATT	0.0252	− 0.179	0.314	− 0.0748	− 0.0276
	(0.13)	(− 0.93)	(1.55)	(− 0.39)	(− 0.13)
LSATN	0.184	− 0.164	0.0827	0.0202	− 0.156
	(1.02)	(− 0.95)	(0.43)	(0.11)	(− 0.84)
TOTCHANGE	0.151	− 0.197	0.619	0.324	1.099**
	(0.33)	(− 0.39)	(1.67)	(0.87)	(3.04)
NETCHANGE	0.442	− 0.165	0.0603	− 0.0314	0.446
	(1.35)	(− 0.50)	(0.18)	(− 0.09)	(1.33)
CHANGE	0.164	0.0186	− 0.226	0.111	− 0.123
	(0.71)	(0.08)	(− 0.98)	(0.48)	(− 0.54)

*T* statistics with standard deviation in parentheses. *: 0.05 **: 0.01 ***: 0.001

**Table 8:** *t* tests for statistically significant differences between question order #1 (**now, then, plot**) and other question orders

NTP	NPT	PNT	PTN	TNP	TPN
LSATT	− 0.0252	− 0.204	0.288	− 0.1000	− 0.0528
	(− 0.13)	(− 1.06)	(1.42)	(− 0.51)	(− 0.25)
LSATN	− 0.184	− 0.348	− 0.101	− 0.164	− 0.340
	(− 1.02)	(− 1.93)	(− 0.51)	(− 0.88)	(− 1.77)
TOTCHANGE	− 0.151	− 0.347	0.468	0.174	0.949*
	(− 0.33)	(− 0.63)	(1.07)	(0.39)	(2.20)
NETCHANGE	− 0.442	− 0.607	− 0.382	− 0.473	0.00390
	(− 1.35)	(− 1.87)	(− 1.16)	(− 1.45)	(0.01)
CHANGE	− 0.164	− 0.145	− 0.390	− 0.0529	− 0.287
	(− 0.71)	(− 0.62)	(− 1.64)	(− 0.22)	(− 1.22)

*t* statistics with standard deviation in parentheses. *: 0.05 **: 0.01 ***: 0.001

**Table 9:** *t* tests for statistically significant differences between question order #2 (**plot, now, then**) and other question orders

PNT	NTP	NPT	PTN	TNP	TPN
LSATT	0.204	0.179	0.492*	0.104	0.151
	(1.06)	(0.93)	(2.53)	(0.56)	(0.75)
LSATN	0.348	0.164	0.247	0.184	0.00847
	(1.93)	(0.95)	(1.27)	(1.03)	(0.05)
TOTCHANGE	0.347	0.197	0.816	0.521	1.296**
	(0.63)	(0.39)	(1.70)	(1.08)	(2.73)
NETCHANGE	0.607	0.165	0.225	0.133	0.610
	(1.87)	(0.50)	(0.68)	(0.41)	(1.84)
CHANGE	0.145	− 0.0186	− 0.245	0.0920	− 0.142
	(0.62)	(− 0.08)	(− 1.05)	(0.40)	(− 0.62)

*T* statistics with standard deviation in parentheses. *: 0.05 **: 0.01 ***: 0.001

**Table 10:** *t* tests for statistically significant differences between question order #1 (**plot, then, now**) and other question orders

PTN	PNT	NTP	NPT	TNP	TPN
LSATT	− 0.492*	− 0.288	− 0.314	− 0.388*	− 0.341
	(− 2.53)	(− 1.42)	(− 1.55)	(− 1.97)	(− 1.61)
LSATN	− 0.247	0.101	− 0.0827	− 0.0625	− 0.238
	(− 1.27)	(0.51)	(− 0.43)	(− 0.31)	(− 1.16)
TOTCHANGE	− 0.816	− 0.468	− 0.619	− 0.294	0.480
	(− 1.70)	(− 1.07)	(− 1.67)	(− 0.86)	(1.46)
NETCHANGE	− 0.225	0.382	− 0.0603	− 0.0917	0.386
	(− 0.68)	(1.16)	(− 0.18)	(− 0.28)	(1.15)
CHANGE	0.245	0.390	0.226	0.337	0.103
	(1.05)	(1.64)	(0.98)	(1.43)	(0.44)

*t* statistics with standard deviation in parentheses. *: 0.05 **: 0.01 ***: 0.001

**Table 11:** *t* tests for statistically significant differences between question order #1 (**then, now, plot**) and other question orders

TNP	PTN	PNT	NTP	NPT	TPN
LSATT	0.388*	− 0.104	0.1000	0.0748	0.0472
	(1.97)	(− 0.56)	(0.51)	(0.39)	(0.23)
LSATN	0.0625	− 0.184	0.164	− 0.0202	− 0.176
	(0.31)	(− 1.03)	(0.88)	(− 0.11)	(− 0.92)
TOTCHANGE	0.294	− 0.521	− 0.174	− 0.324	0.775*
	(0.86)	(− 1.08)	(− 0.39)	(− 0.87)	(2.32)
NETCHANGE	0.0917	− 0.133	0.473	0.0314	0.477
	(0.28)	(− 0.41)	(1.45)	(0.09)	(1.43)
CHANGE	− 0.337	− 0.0920	0.0529	− 0.111	− 0.234
	(− 1.43)	(− 0.40)	(0.22)	(− 0.48)	(− 1.01)

*t* statistics with standard deviation in parentheses. *: 0.05 **: 0.01 ***: 0.001

**Table 12:** *t* tests for statistically significant differences between question order #1 (**then, plot, now**) and other question orders

TPN	TNP	PTN	PNT	NTP	NPT
LSATT	− 0.0472	0.341	− 0.151	0.0528	0.0276
	(− 0.23)	(1.61)	(− 0.75)	(0.25)	(0.13)
LSATN	0.176	0.238	− 0.00847	0.340	0.156
	(0.92)	(1.16)	(− 0.05)	(1.77)	(0.84)
TOTCHANGE	− 0.775*	− 0.480	− 1.296**	− 0.949*	− 1.099**
	(− 2.32)	(− 1.46)	(− 2.73)	(− 2.20)	(− 3.04)
NETCHANGE	− 0.477	− 0.386	− 0.610	− 0.00390	− 0.446
	(− 1.43)	(− 1.15)	(− 1.84)	(− 0.01)	(− 1.33)
CHANGE	0.234	− 0.103	0.142	0.287	0.123
	(1.01)	(− 0.44)	(0.62)	(1.22)	(0.54)

*t* statistics with standard deviation in parentheses. *: 0.05 **: 0.01 ***: 0.001

## Appendix 3: Y-axis labelled ‘low’ to ‘high’ versus 1–10

Table 13 shows the average response by year plotted by respondents in the sub-sample presented with a y-axis labelled 1–10 compared to those presented with a y-axis labelled “low” to “high”. There is no statistically significant difference.

**Table 13:** Comparison of responses between scaled and unscaled plots in sample 2

GROUP	T1	T2	T3	T4	T5	T6	T7	T8	T9	T10
Full Online Sample	6.3	6.4	6.4	6.4	6.3	6.3	6.2	6.3	6.4	6.5
Unscaled Y-axis	6.3	6.4	6.4	6.4	6.3	6.2	6.2	6.3	6.4	6.5
Scaled Y-axis	6.4	6.4	6.5	6.4	6.3	6.3	6.3	6.4	6.6	6.6

## Footnotes

1 It is an open question whether life satisfaction is latent, like intelligence, or constructed in response to a question, like an opinion. For ease of graphical representation, the discussion here assumes that latent life satisfaction exists and can be quantified, at least ordinally.

2 Net change could also be represented as t_10_ − t_1_, but 76 respondents in the student sample drew narrow plots and thus had missing values for some early and/or late time periods when the 10 × 10 transparency was overlaid onto their graphs. The specification of net change above overcomes this problem without requiring the imputation of any values.

3 To reduce well-established concerns around framing and priming affecting life satisfaction responses (Bertrand & Mullainathan 2001), each question in the online survey was preceded by a benign question such as how often the individual skips breakfast or whether they are a night owl or morning person.
